# Locally epistatic models for genome-wide prediction and association by importance sampling

**DOI:** 10.1186/s12711-017-0348-8

**Published:** 2017-10-17

**Authors:** Deniz Akdemir, Jean-Luc Jannink, Julio Isidro-Sánchez

**Affiliations:** 1 StatGen Consulting, Ithaca, NY USA; 2 Robert W. Holley Center for Agriculture and Health, USDA-ARS, Ithaca, NY USA; 3 Department of Animal and Crop Science, University College Dublin, Dublin, Ireland

## Abstract

**Background:**

In statistical genetics, an important task involves building predictive models of the genotype–phenotype relationship to attribute a proportion of the total phenotypic variance to the variation in genotypes. Many models have been proposed to incorporate additive genetic effects into prediction or association models. Currently, there is a scarcity of models that can adequately account for gene by gene or other forms of genetic interactions, and there is an increased interest in using marker annotations in genome-wide prediction and association analyses. In this paper, we discuss a hybrid modeling method which combines parametric mixed modeling and non-parametric rule ensembles.

**Results:**

This approach gives us a flexible class of models that can be used to capture additive, locally epistatic genetic effects, gene-by-background interactions and allows us to incorporate one or more annotations into the genomic selection or association models. We use benchmark datasets that cover a range of organisms and traits in addition to simulated datasets to illustrate the strengths of this approach.

**Conclusions:**

In this paper, we describe a new strategy for incorporating genetic interactions into genomic prediction and association models. This strategy results in accurate models, with sometimes significantly higher accuracies than that of a standard additive model.

**Electronic supplementary material:**

The online version of this article (doi:10.1186/s12711-017-0348-8) contains supplementary material, which is available to authorized users.

## Background

The genetic basis and evolutionary causes of quantitative variation were first proposed at the end of the nineteenth century [1, 2]. The statistical tools developed (correlation and regression) were the foundation of biometry science. Considerable efforts were made to identify the genetic architecture of traits by mapping quantitative trait loci (QTL) in humans, animals and plants [3–5]. Quantitative genetic theory focuses on finding the underlying genetic variation in genes by applying the classical infinitesimal (polygenic) model [2, 6].

In the infinitesimal model, the genetic values of individuals are assumed to be generated by an infinite number of unlinked and non-epistatic genes, each with an independent infinitesimal effect. In this model, quantitative genetics focuses on the additive effects of individual alleles. The rate of change of a trait and the genotypic variance depend primarily on additive effects, hence interaction terms are often neglected. However, while for many QTL, thousands of studies have been carried out, few examples that have successfully exploited mapped QTL have been reported in the literature [7]. Indeed, although genome-wide association studies (GWAS) have discovered hundreds of single nucleotide polymorphism (SNPs) significantly associated with complex traits [8–12], they have explained only a small proportion of the estimated genetic variation, a term coined “missing heritability” [13]. Using SNP data to detect loci with a large effect by associating common phenotypes with common genotypes, provides a way to capture the infinitesimal effects. In addition, the genome-wide predictive models, which are mainly used in genomic selection of animals or plants showed that the results from models that assume additive infinitesimal effects can be accurate and informative [14]. The infinitesimal model has very powerful simplifying statistical properties and avoids the need to specify individual gene effects [15]. Association studies that involve complex interactions between loci are complicated by the large number of effects that need to be tested simultaneously. Most GWAS studies and prediction models focus on estimating the effects of each marker and lower level interactions [16]. For a dataset of *m* markers, a genome-wide analysis with two loci will involve evaluating a number of possibilities of the order of $m^2$ and *m* can easily exceed millions. Because of the consequent multiple testing burden, the methods used to identify and model epistasis lack statistical power, and they are computationally exhaustive or even unfeasible. Several factors make it difficult to estimate the true numbers and the effects of loci that influence a QTL. The detection of epistasis is a key factor for explaining the “missing heritability” [17]. The form and strength of epistasis that we expect for QTL depend crucially on the specific details of gene action. Gene interactions are important because (1) they cause the additive effects of alleles to change as the genetic composition of the population changes [18] and (2) they might also slow down selection response, because alleles might only become favorable as the genetic background changes during selection [19]. A common approach to identify interactions is to test SNPs with the most significant additive effects. This approach can be problematic since the absence of additive effects might be generated by interacting loci. In this article, we propose a new hybrid (machine learning + mixed models) approach that (1) results in a flexible class of models that can be used to capture additive, locally epistatic genetic effects, and gene $\times$ background interactions, and (2) allows to incorporate one or more annotations into the genomic prediction and association models. Thus, the main aim of this study was to measure and incorporate additive and local epistatic genetic contributions to complex traits.

## Methods

### Materials

The genetic material used in this study to compare our novel prediction and association models to the standard linear genomic BLUP (GBLUP) model consists of four different datasets on maize, rice, wheat and mouse (Table 1). The maize dataset which was used in previous studies [22, 23] was downloaded from panzea.org. The rice dataset can be downloaded from www.ricediversity.org and was used in [24–26]. The wheat dataset was downloaded from the triticale toolbox dataset www.triticaletoolbox.org and the mouse dataset, published in [27], was accessed from the “synbreeddata” package [28] available in R [29]. To determine if the locally epistatic rules (LER) model can be used to locate interacting loci and to compare its results to those from a standard additive mixed modeling approach, we devised the following experiment. We simulated 1000 independent SNPs that were coded 0, 1 and 2 for 2000 individuals. Five genetic effects $g_i, i=1,\ldots , 5$ at five loci were generated according to the formulas in Table 2. Each effect was standardized to have a variance of 1 across the genotypes and the total genotypic value of a genotype was calculated as the sum of these effects. Each of these quantitative trait loci involved three closely located SNPs. Effect $g_1$ was completely additive, while the other effects contained SNP by SNP interactions, SNP by background interactions or both. The formula for each of these effects are in Table 2. The individuals were evenly assigned to one of the two sexes at random, which in turn was reflected in the genetic values as a fixed difference of 5 units. The final phenotypes for the individuals were obtained by adding independent and identically distributed, zero centered normal random variables to genetic values to obtain a broad-sense heritability of 2/3. In this simulation study, we have partitioned the genome into 10 segments for the LER model. The whole experiment was replicated 100 times to obtain the results in Table 3.

**Table 1:** Summary of the features of the datasets and the hyper-parameter settings for the results presented in Fig. 5

Dataset	Number of individuals	Number of SNPs	Traits	Mean depth	Nrules	Nsplits	Proprow	Propcol
Rice	299	73K	PH, FLW, LG, GRL, GRW, 1000GW, YLD	4	500	5	.3	.1
Mouse	1940	12K	Body weight, growth slope		2000	10	.1	.05
Maize	4676	125K	GDD_DTS	2	1000	10	.1	.05
			GDD_DTA, GDD_ASI, DTS	2	200	40 (using hotspots)	.1	.05
			DTA, ASI, PH, EH					
			PH.EH, EHdivPH, PHdivDTR					
Wheat	337	3355	FD, PMD, PH, YLD, WGP	1	500	2	.3	.1
			HD, WAX					

Trait Names: *GDD_DTA* growing degree days to silk, *GDD_DTA* growing degree days to anthesis, *GDD_ASI* growing degree days to anthesis-silking interval, *DTS* days to silking, *DTA* days to anthesis, *ASI* anthesis silking interval days, *PH* plant height, *EH* ear height, *PH-EH* PH minus EH, *EHdivPH* EH divided by PH, *PHdivDTR* PH divided by days to anthesis *FLW* flag leaf width, *LG* lodging, *GRL* grain length, *GRW* grain weight, *1000GW* thousand grain weight, *YLD* yield, *FD* flowering day, *PMD* physiological maturity day, *WGP* whole grain protein, *HD* heading date Julian, *WAX* waxiness

**Table 2:** Definition of five genetic effects used in simulations to determine if the LER model could locate interacting loci

Effect	
$$g_1=(.6*x_{8}+.5*x_{11}-.4*x_{14})$$	
$$if (pc_1<0) \left[ g_2=.6*x_{208}-.5*x_{211}-.4*x_{214}\right]$$	
else $\left[ g_2=-(.6*x_{208}+.5*x_{211}+.4*x_{214})\right]$	
$$g_3=(.6*x_{408}+.5*x_{411}-.4*x_{414})^2$$	
$$if(pc_1<0) \left[ g_4=((.6*x_{608}+.5*x_{611}-.4*x_{614})^2)\right]$$	
else $\left[ g_4=(-(.6*x_{608}-.5*x_{611}+.4*x_{614})^2)\right]$	
$$if(pc_1<0) \left[ g_5=((.6*x_{808}+.5*x_{811}-.4*x_{814}+.5*pc_2)^2)\right]$$	
else $\left[ g_5=((-.6*x_{808}-.5*x_{811}-4*x_{814}+.5*pc_2)^2)\right]$	

Five genetic effects $g_i, i=1,\ldots , 5$ at five loci were generated according to the formulas below. Each effect was standardized to have a variance of 1 over the simulated genotypes and the total genotypic value of an genotype was calculated as the sum of these effects. The individuals were evenly assigned to one of the two sexes at random, which in turn was reflected in the genetic values as a fixed difference of 5 units. The final phenotypes for the individuals were obtained by adding independent and identically distributed, zero centered normal random variables to genetic values to obtain a broad-sense heritability of 2/3

**Table 3:** Number of times the true loci are recovered by standard GWAS and LER over 100 repetitions of the simulated association experiment described in Table 2

Model/marker	$x_{8}$	$x_{11}$	$x_{14}$	$x_{208}$	$x_{211}$	$x_{214}$	$x_{408}$	$x_{411}$	$x_{414}$	$x_{608}$	$x_{611}$	$x_{614}$	$x_{808}$	$x_{811}$	$x_{814}$
GWAS	73	77	75	9	71	71	83	63	0	26	65	29	74	77	34
LER	93	97	100	100	100	100	100	100	20	100	97	68	100	99	57

**Table 4:** A scenario which shows an interaction pattern between two markers generated by a simple rule “$I(m1<2)*I(m2>1)\rightarrow$ – else +”

Genotype–phenotype				Allele coding and m1*m2			
m1/m2	BB	Bb	bb	m1/m2	0	1	2
AA	+	+	+	0	0	0	0
Aa	–	+	+	1	0	1	2
aa	–	+	+	2	0	2	4

The standard multiplicative formulation ($m1\times m2$) cannot adequately represent this interaction and other terms would be needed in the model (additive, additive $\times$ additive, additive $\times$ dominance, dominance $\times$ dominance, see factorial model in [59, 60])

### Methods

**Fig. 1:**
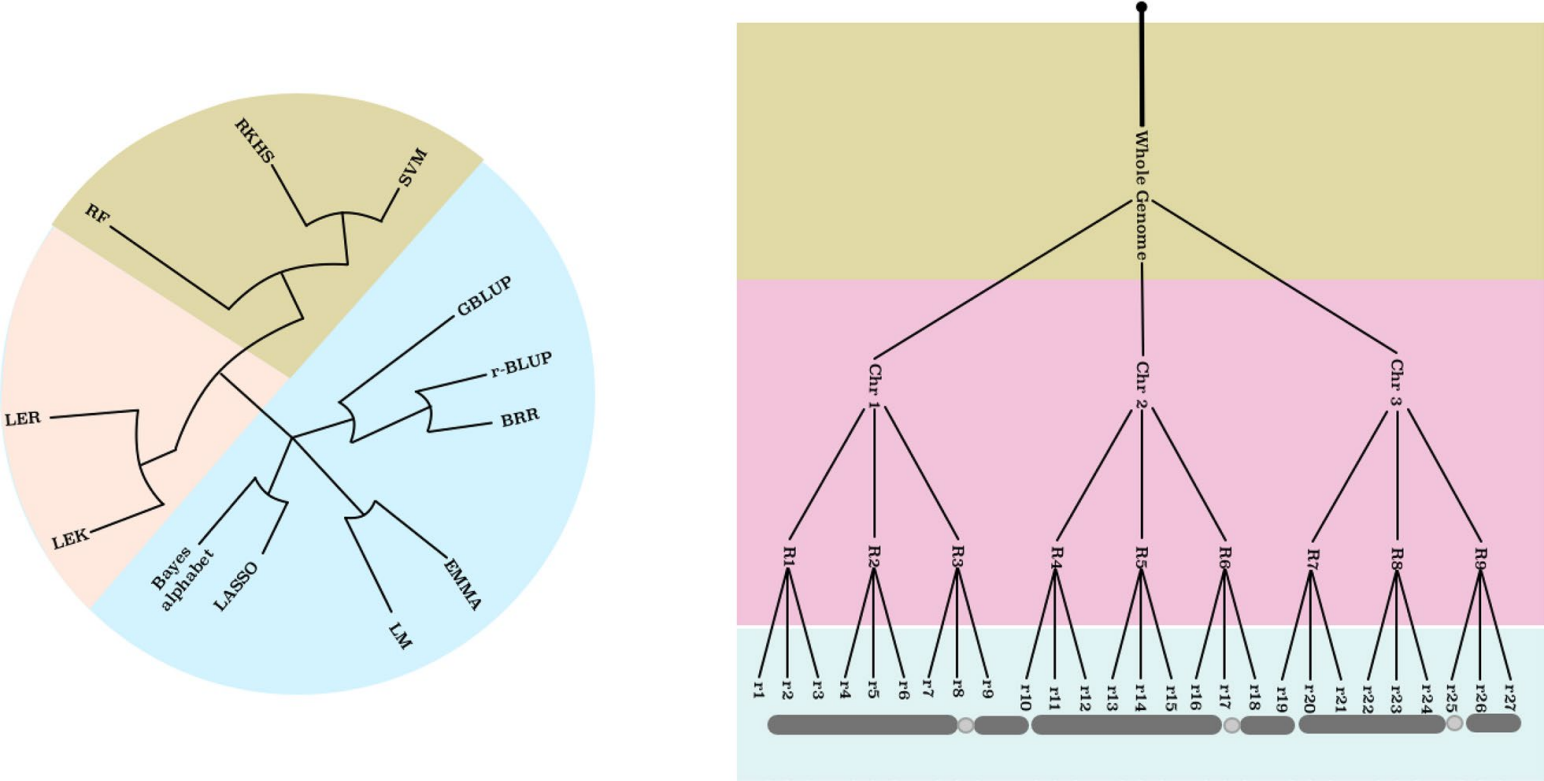
Many of the models used in genomic prediction and association analyses are additive: These include ridge regression-best linear unbiased prediction (rr-BLUP) [31, 32], Lasso [33], Bayesian–Lasso [34], Bayesian ridge regression, Bayesian alphabet [35, 36], GBLUP and EMMA [47]. Several scientists have also developed methods to use genome-wide epistatic effects: RKHS [37, 38]), RF [39], SVM. The dendrogram on the left was obtained based on a table of the properties of different models, this table included variables such as “additive-epistatic”, “global-local”, “marker-kernel based”; it should not be taken as a formal clustering of models. The colors attached to the groups in the dendrogram are matched with different parts of the genome to illustrate the focus of each of these groups. Locally epistatic kernels (LEK) and locally epistatic rules (LER) models that use local epistasis

In statistical genetics, an important task involves building predictive and association models of the genotype–phenotype relationship to attribute a proportion of the total phenotypic variance to the variation in genotypes. There are numerous statistical models used in genomic prediction and association (see Fig. 1). An evaluation of these methods for predicting quantitative traits is in [30]. Many of the models used for genomic prediction and association are additive. These include ridge regression-best linear unbiased prediction (rr-BLUP) [31, 32], Lasso [33], Bayesian–Lasso [34], Bayesian ridge regression, Bayesian alphabet [35, 36]), GBLUP. Some methods for capturing genome-wide epistasis include the reproducing kernel Hilbert spaces regression (RKHS) approach [37, 38] and related support vector machine regression or partitioning based on random forest [39]. These models can be used to predict genetic values but do not provide satisfactory information about the genetic architecture of traits. An alternative approach when studying epistasis is to consider only local epistasis [40], i.e., only epistatic interactions between closely located loci. It is reasonable to assume that only the epistatic effects that arise from alleles in gametic disequilibrium, between closely located loci can contribute to long-term response since recombination disrupts allelic combinations that have specific epistatic effects. In a recent article [40], we proposed a modeling approach that uses RKHS-based approaches to extract locally epistatic effects, which we referred to as the locally epistatic kernels (LEK) model. It was shown in [40] that LEK models could be used to improve prediction accuracies and provide useful information about genetic architecture.

### Importance sampling learning ensembles and rule ensembles

The LER models introduced here uses the importance sampling learning ensembles (ISLE) [41] based rule extraction procedures for genomic prediction and GWAS. We described and illustrated the use of ISLE-based approaches with genomic data in [42]. Nevertheless, for completeness, we include a description of ISLE-based ensemble model generation procedure in this section. Given a learning task and a relevant dataset, we can generate an ensemble of models from a predetermined model family; an ensemble of models is a single model that combines this ensemble of models. The main discovery is that ensemble models are much more accurate than the individual models that make them up when the individual members of the ensemble are accurate (low bias) and diverse (high variance). Ensemble models are shown to perform extremely well in a variety of scenarios and to have desirable statistical properties. Members of the ensemble are generated by fitting models from the chosen family to perturbed data. For instance, bagging [43] bootstraps the training dataset and produces a model for each bootstrap sample. Random forest [39, 44] creates models by randomly selecting a subset of observations and / or explanatory variables while generating each model. Boosting is a bias-reduction technique, AdaBoost iteratively builds models by varying case weights and using the weighted sum of the estimates of the sequence of models. There have been attempts to unify these ensemble learning methods. One such framework is the ISLE. Let us assume that we want to generate an ensemble of models for predicting the continuous outcome variable *y* from vector **p** of input variables $\varvec{x}$ from a model family $\mathcal {F}=\{f(\varvec{x}, {{\varvec{\theta }}}): {{\varvec{\theta }}}\in \ominus\}$ indexed by the parameter ${{\varvec{\theta }}}.$ The final ensemble model produced by the ISLE framework has an additive form:

1 $$\begin{aligned} F(\varvec{x})=w_0+\sum _{j=1}^{\textit{M}}w_{j} f(\varvec{x}, {{\varvec{\theta }}}_j) \end{aligned}$$

where $\{f(\varvec{x}, {{\varvec{\theta }}}_j)\}_{j=1}^{\textit{M}}$ are base learners selected from $\mathcal {F}.$ A two-step approach is used to produce $F(\varvec{x})$. The first step involves sampling the space of possible models to obtain $\{\widehat{{{\varvec{\theta }}}}_j\}_{j=1}^{\textit{M}}$. The second step involves combining the base learners by choosing weights $\{w_j\}_{j=0}^{\textit{M}}$ in Eq. (1). The pseudo code to produce $\textit{M}$ models $\{f(\varvec{x}, \widehat{{{\varvec{\theta }}}}_j)\}_{j=1}^{\textit{M}}$ under the ISLE framework is given in Algorithm 1: 
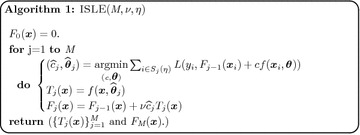


Here, *L*(., .) is a loss function; $S_j(\eta )$ is a subset of the indices $\{1,2,\ldots , n\}$ chosen by a sampling scheme $\eta ,$ and $0\le \nu \le 1$ is a memory parameter. The classic ensemble methods of bagging, random forest, AdaBoost, and gradient boosting are special cases of the ISLE ensemble model generation procedure [45]. In bagging and random forests, the weights in Eq. (1) are set to predetermined values, i.e. $w_0=0$ and $w_j=\frac{1}{\textit{M}}$ for $j=1,2,\ldots ,\textit{M}.$ Boosting calculates these weights in a sequential fashion at each step by having positive memory $\nu ,$ estimating $c_j$ and takes $F_\textit{M}(\varvec{x})$ as the final prediction model. Friedman and Popescu [41] recommend learning the weights $\{w_j\}_{j=0}^{\textit{M}}$ using Lasso [33]. Let $\mathbf T ={\left( T_j(\varvec{x}_i) \right) _{i=1}^n}_{j=1}^{\textit{M}}$ be the $n\times \textit{M}$ matrix of predictions for the *n* observations by the $\textit{M}$ models in an ensemble. The weights $(w_0,\varvec{w}=\{w_m\}_{m=0}^{\textit{M}})$ are obtained from:

2 $$\begin{aligned} \hat{\varvec{w}}=\underset{\varvec{w}}{{\text {argmin}}} (\varvec{y}-w_0\varvec{1}_n-T\varvec{w})'(\varvec{y}-w_0\varvec{1}_n-T\varvec{w})+\lambda \sum _{m=1}^{\textit{M}}|w_m|. \end{aligned}$$

$\lambda >0$ is the shrinkage operator, larger values of $\lambda$ decrease the number of models included in the final prediction model. The final ensemble model is given by:

3 $$\begin{aligned} \hat{F}(\varvec{x})=\hat{w}_0+\sum _{m=1}^{\textit{M}}\hat{w}_{m} T_m(\varvec{x}). \end{aligned}$$

The ISLE approach produces a generalized additive model (GAM) [46]. A few other post-processing approaches such as partial least squares regression, multivariate kernel smoothing, and weighting, as well as the use of rules in semi-supervised and unsupervised learning, are described in [42]. There is no restriction on the choice of the family of base learners, $\mathcal {F},$ in the ISLE procedure. The most popular choice for the base learners is the class of regression and classification trees. Tree-based methods have the advantage of being virtually assumption free, they are simple to fit and interpret. They can capture interactions and handle missing values by using surrogate splits. In addition, trees can naturally handle all types of input variables, i.e., numeric, binary, categorical. They are invariant under monotone transformations and scaling of the variables. Trees have a high variance on these data due to the correlation in the predictors. An ensemble of tree models succeeds in smoothing out this variance and hence reduces test error.

**Fig. 2:**
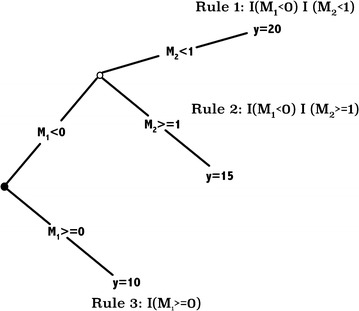
An example of a tree to rules. At each intermediate node, an observation goes to the left branch if and only if the condition shown there is satisfied. A simple regression tree which can be represented as $y=20 I(M_1<0)(M_2<1)+15 I(M_1<0)I(M_2\ge 1)+10 I(M_1\ge 0).$ Each leaf node defines a rule which can be expressed as a product of indicator functions of half spaces. Each rule specifies a ‘simple’ rectangular region in the input space

A tree with *K* terminal nodes defines a *K* partition of the input space where the membership to a specific node, say node *k*, can be determined by applying the conjunctive rule $r_k(\varvec{x})=\prod\nolimits_{l=1}^{p}I(x_l\in s_{lk}),$ where *I*(.) is the indicator function, $\varvec{x}=(x_1,x_2,\ldots , x_p)$ are the input variables. The regions $s_{lk}$ are intervals for continuous variables and a subset of the possible values for a categorical variables. The easiest way to create an ensemble of rules is to extract it from an ensemble of decision trees. In a tree, each path from the root node to a leaf defines a rule. An example of a regression tree and the corresponding rules extracted from this tree are displayed in Fig. 2. The complexity of trees or rules (the degree of interactions between the input variables) in the ensemble increases as the number of nodes increases from the root to the final node (depth). Individual trees can be pruned using a cost complexity criterion. For example, a popular cost complexity criterion [48] that balances the residual sum of squares and the complexity of the tree can be written as:

$$\begin{aligned} CCC(T) := RSS(T) + cp |nodes(\widehat{y})| \end{aligned}$$

where *T* is a regression tree, $cp \ge 0$ the complexity parameter/regularization parameter, $|nodes(\widehat{T})|$ denotes the number of nodes of tree $\widehat{y},$ and *RSS*(*T*) is the residual sum of squares of the tree. In addition, the parameters **minbucket**, **minsplit**, and **maxdepth** constrain the solution to a minimum number of observations in each terminal node, a minimum number of observations in each internal node, and a maximum tree-depth. There are numerous options for building tree models: these include iterative dichotomiser 3 (ID3) [49], C4.5 [50], classification and regression trees (CART) [48], etc. In this paper, the model $f(\mathbf X )$ in Eq. (6) at each iteration of the EM algorithm was extracted by the CART approach using the R package **rpart** [51]. Suppose an ensemble of tree models was generated by the ISLE algorithm in Algorithm 1 and let $\mathbf{R}={\left( r_k(\varvec{x}_i) \right) _{i=1}^n}_{k=1}^K$ be the $n\times K$ matrix of rules derived from this ensemble of trees. The **rulefit **algorithm of Friedman and Popescu [52] uses the weights $(w_0,\varvec{w}=\{ {w_k}\}_{k=0}^{K})$ that are estimated from:

4 $$\begin{aligned} \hat{\varvec{w}}=\underset{\varvec{w}}{{\text {argmin}}} (\varvec{y}-w_0\varvec{1}_n-\mathbf{R}\varvec{w})'(\varvec{y}-w_0\varvec{1}_n-\mathbf{R}\varvec{w})+\lambda \sum _{k=1}^K|w_k| ,\end{aligned}$$

in the final prediction model:

5 $$\begin{aligned} \hat{F}(\varvec{x})=\hat{w}_0+\sum _{k=1}^{K}\hat{w}_{k} r_k(\varvec{x}). \end{aligned}$$

### Locally epistatic models via rules (LER)

When applying the ISLE approach to genomic data special considerations need to be taken into account because of the dependencies among features such as the arrangement (localization, spacing and number) of SNPs on chromosomes, the linkage between SNPs, or annotations that put certain SNPs in the same groups. Joint consideration of linkage and epistasis is a necessary step for the models that incorporate the interactions for more than one locus. Complex systems that use evolutionary mechanisms such as selection proportional to fitness, recombination and mutation tend to generate short adapted and specialized structures the number of which will increase exponentially in successive generations. For instance, the scheme theorem of Holland [53] can be stated as:

$$\begin{aligned} N(H, t+1)&=N(H, t) *(1+c) *(1-Pr(H \text { is lost due to recombination})) \\&*(1- Pr(H \text { is lost to mutation})), \end{aligned}$$

where *N*(*H*, *t*) is the frequency of a haplotype (schema) *H* at time *t,* and *c* the relative fitness of *H* compared to all other haplotypes in the population. It can be argued that $Pr(\text {H is lost due to recombination})$ is an increasing function of the linkage length of *H* and $Pr(\text {H is lost to mutation})$ is an increasing function of the order of *H*, i.e., number of loci in *H* that affect its fitness. The epistatic effects involving unlinked loci have a high probability of being lost due to recombination and will not contribute to the subsequent response. This argument forms the basis of the “building blocks” hypothesis in the evolutionary theory. Because of these considerations that are unique to genomic data, we propose a modification of the ISLE algorithm so that the interactions among genes are restricted to local genomic regions. Locally epistatic rule-based model fitting starts with a definition of genomic regions; suppose we defined *k* such regions. Region definition is followed by the extraction of local rules from each genomic region $j=1,2,\ldots , k.$ using the ISLE algorithm. The rules are extracted from trees that predict the estimated genetic value from SNPs in the region. Since the rules are independently generated for each region, this step can be computationally accomplished in parallel without loading all the genetic data to computer memory. The values of the rules from all regions are calculated for the *n* training individuals, they are standardized with respect to their sample standard deviation and combined in a matrix $n\times r$ matrix $\mathbf R .$ The model behind the proposed mixed effects regression tree method is:

6 $$\begin{aligned}\mathbf{y}=f(\mathbf X )+\mathbf Z \mathbf{g}+\mathbf{e}, \end{aligned}$$

$\mathbf{g}\sim N(\varvec{0},\sigma ^{2}_{g} \mathbf G ),$ $\mathbf{e}\sim N(\varvec{0},\sigma ^2\mathbf I _{m}),$ where all quantities are defined as in a classical linear mixed effects model except that a more general and unspecified fixed part, $f(\mathbf X ),$ which describes the vector obtained by applying the function *f* to each row of $\mathbf X ,$ now replaces the usual linear part $\mathbf X {{\varvec{\beta }}}$ which will be estimated with a single tree. The random part, $\mathbf{Z }\mathbf{g},$ is still assumed linear with a covariance structure given by $\sigma ^{2}_{g} \mathbf G ,$ where $\mathbf G$ is an additive genetic similarity matrix. Given $\mathbf M ,$ the marker allele frequency centered incidence matrix, the matrix $\mathbf G$ can be calculated as $\mathbf G = \mathbf M {} \mathbf M '/k$ where *k* is the sum of the variances of the SNPs [54]. The ML-based EM-algorithm to fit this model is described in [55] and [56]. As with the mixed model association methods, the aim of including a random term that accounts for the genetic structural effects is to correct for confounding between the familial effects and the effects of the loci. The second step in locally epistatic rule based model fitting is the post-processing step where we obtain a final prediction model using the extracted rules as input variables. The mixed models (MM) methodology has a special place in quantitative genetics because it provides a formal way of partitioning the variability observed in traits into heritable and environmental components, it is also useful to control for population structure and relatedness for GWAS. In a mixed model, genetic information in the form of a pedigree or SNP data can be used in the form of an additive genetic similarity matrix that describes the similarity based on additive genetic effects (GBLUP). For the $n\times 1$ response vector $\mathbf{y},$ the GBLUP model can be expressed as:

7 $$\begin{aligned} \varvec{y}=\mathbf X {{\varvec{\beta }}}+\mathbf Z \varvec{g}+\varvec{e},\end{aligned}$$

where $\mathbf X$ is the $n\times p$ design matrix for the fixed effects, ${{{\varvec{\beta }}}}$ is a $p\times 1$ vector of fixed effects, $\mathbf Z$ is the $n\times q$ design matrix for the random effects; the vectors of random effects $\mathbf{g}$ and $\mathbf{e}$ are assumed to be independent multivariate normal (MVN) random variables with means $0$ and corresponding covariances $\sigma ^{2}_{g} \mathbf G$ and $\sigma ^{2}_{e} \mathbf I _{n}$ where $\mathbf G$ is the $q\times q$ additive genetic similarity matrix. It is known that model in Eq. (7) is equivalent to a MM in which the additive marker effects are estimated via the following model (rr-BLUP):

8 $$\begin{aligned} \mathbf{y}=\mathbf X {{\varvec{\beta }}}+\mathbf Z {} \mathbf M \mathbf{u}+\mathbf{e}, \end{aligned}$$

where $\mathbf X$ is the $n\times p$ design matrix for the fixed effects, ${{\varvec{\beta }}}$ is a $p\times 1$ vector of fixed effect coefficients, $\mathbf Z$ is the $n\times q$ design matrix for the random effects $\mathbf M$ is $q\times m$ marker allele frequency centered incidence matrix; $\mathbf{u}$ and $\mathbf{e}$ are assumed to be independent MVN random variables with means $0$ and corresponding covariances $\sigma ^{2}_{u} \mathbf{I}_m$ and $\sigma ^{2}_{e} \mathbf I _{n}.$ The conversion between the predicted genotypic values $\widehat{\varvec{g}}$ in Eq. (7) and the predictions for marker effects $\widehat{\varvec{u}}$ in Eq. (8) are given by:

9 $$\begin{aligned} \widehat{\varvec{u}}=\mathbf M '{} \mathbf Z '(\mathbf Z {} \mathbf M {} \mathbf M '{} \mathbf Z ')^{-1}\widehat{\varvec{g}}. \end{aligned}$$

In this article, we use the rr-BLUP model for post-processing the rules:

10 $$\begin{aligned} \mathbf{y}=\mathbf X {{\varvec{\beta }}}+\mathbf Z \mathbf R \varvec{\alpha }+\mathbf{e}, \end{aligned}$$

where $\mathbf Z$ is a $n\times q$ design matrix for the random effects, $\mathbf R$ is a $q\times r$ design matrix for the centered and scaled rules, and $(\varvec{\alpha }',\varvec{e}')'$ follows a MVN distribution with mean $0$ and covariance ,

$$\begin{aligned} \left( \begin{array}{cc} \sigma _{{\alpha }}^2 \mathbf I _{r} &{} \varvec{0}\\ \varvec{0}&{} \sigma ^2_e \mathbf I _{n} \end{array} \right) . \end{aligned}$$

Note that each rule is a function of the SNPs. Using estimated coefficients, $\widehat{\varvec{\alpha }},$ we calculate the estimated genotypic value for an individual with SNPs $\varvec{m}$ as $\widehat{R(\varvec{m})}\widehat{\varvec{\alpha }}$ where $R(\varvec{m})=(R_1(\varvec{m}),R_2(\varvec{m}),\ldots , R_r(\varvec{m})).$

#### Importance and interaction measures

In addition to having a good prediction performance, a good model should also provide a description of the relationship between the input variables and the response. The rules and the estimated coefficients of the LER model can be used extract several importance and interaction measures. Let $I(m_\ell \in R_j)$ denote the indicator function for the inclusion of SNP $\mathbf M _\ell$ in rule $R_j.$

- Since $\mathbf R$ has standardized columns, $|\widehat{\varvec{\alpha }}|$ can be used as importance scores for the rules in the model.
- A measure of importance for a SNP $\ell$ is obtained by $I_j=\sum\nolimits_{j=1}^{r}|\widehat{\alpha _j}| I(m_\ell \in R_j).$
- A measure of the interaction strength between two SNPs $\ell$ and $\ell '$ is obtained by: $I_{\ell \ell '}=\sum\nolimits_{j=1}^{r}|\widehat{\alpha _j}| I(m_\ell \in R_j)I(m_{\ell '}\in R_j).$
- A measure of the interaction strength between SNPs $\ell _1, \ell _2,\ldots , \ell _l$ is given by $I_{\ell _1 \ell _2\ldots ,\ell _l}=\sum\nolimits_{j=1}^{r}|\widehat{\alpha _j}| \prod\nolimits _{k=\ell _1}^{l}I(m_{\ell _k}\in R_j).$
- Importance of a region: Sum of the rule or marker importances within a region.

The variable importance and interaction measures are in line with the stability selection methods [57, 58]. With stability selection, the data are perturbed (for example by subsampling) many times and the structures or variables that occur in a large fraction of the resulting selection sets are deemed important.

#### “Tuning” the LER algorithm

While fitting the model in Eq. (1), we need to decide on the values of a number of arguments (hyper-parameters) to control the fitting behavior. Hyper-parameter settings can have a strong impact on the prediction accuracy of the trained model. Optimal hyper-parameter settings often differ for different datasets. Therefore, they should be tuned for each dataset. Since the model training process does not set the hyper-parameters, a meta process for tuning the hyper-parameters is needed. Conceptually, hyper-parameter tuning is an optimization task, just like model training. The hyper-parameters in LER models may be selected by comparing the cross-validated accuracies within the training dataset for several reasonable choices. For each proposed hyperparameter setting, the inner model training process comes up with a model for the dataset and outputs evaluation results on hold-out or cross-validation datasets. After evaluating a number of hyperparameter settings by a method like grid or random search, the hyperparameter tuner settings that yield the best performing model are used. The choice of the hyper-parameter for the LER models should also reflect the available resources and the needs. For instance, the number of regions that we can define depends on the number of SNPs and on the resolution that the dataset allows, and a more detailed analysis might only be suitable when the number of SNPs and the number of genotypes in the training dataset are large. The LER methodology provides the user with a range of models with different levels of detail, sparsity, and interactions. The depth of a rule is a hyper-parameter of the LER models since it controls the degree of interaction. A term involving the interaction of a set of variables can only enter the model if there is a rule that splits the input space based on those variables. One way to control the amount of interactions is to grow the trees to a certain depth. We can call this parameter the “maxdepth” parameter. In this article, we allowed different rules to enter the model by setting the “maxdepth” of each tree independently to a random variable generated from a truncated Poisson distribution that turned the parameter into a continuous one which controls the “mean depth” of rules. This allows a diverse set of rules with different depths. The “mean depth” parameter controls the distribution of the complexity of the rules that comprise the ensemble. A choice can be based on the a-priori suspicions about the nature of the target. We also note that the number of rules in a tree is increased by the order of the square of the “mean depth” parameter. The effect of increasing this parameter is a decrease of the “mean depth” of the trees and “mean depth” and the number of rules extracted from each tree. The trees and the associated rules can be pruned during extraction with heuristics such as complexity cost pruning, or reduced error pruning [59]. The hyper-parameters “proprow” and “propcol” control the number of sampled rows and columns from the full data for training an individual tree. Precision of the resulting trees and rules increases whereas their accuracy decreases as either of these parameters decrease. Improving the prediction accuracy of a tree and the precision of its splits is a balancing act; in general, we should aim at having enough examples and a manageable number of SNPs for each run of the tree extraction. “nrules” is a related hyper-parameter that controls the number of rules to be extracted from each genomic region. In order to use all of the training data and to have reliable importance statistics, each genotype and each marker should be sampled several times during the extraction of rules. The detailed steps taken during the model fitting process are provided in an algorithmic form in Algorithm 2:

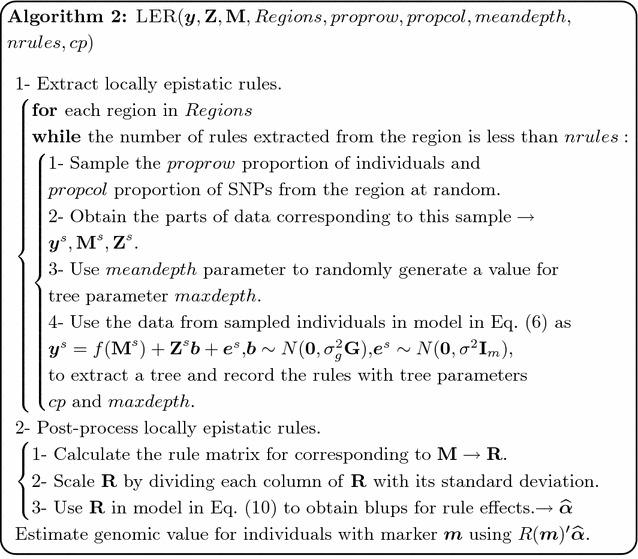



For the maize dataset, we used two settings to split the SNPs into contiguous and non-intersecting regions. In the first setting, each chromosome was split into 10 segments by dividing the chromosome into blocks with approximately the same number of SNPs. In the second setting, we used maize recombination hot-spots [60] to split each maize chromosome into 40 segments. The rules were extracted using the SNP in each region along with the first three principal components (PC) of the genome-wide SNPs. The rice, wheat, and mouse datasets were treated similarly. The details of the settings of the LER algorithm for each dataset are in Table 1. To show that the model is robust over reasonable choices of the hyper-parameter values, we included the results for several hyper-parameter settings in Additional file 1. Additional file 2 provides sample code for replicating the simulated experiment.

## Results

**Fig. 3:**
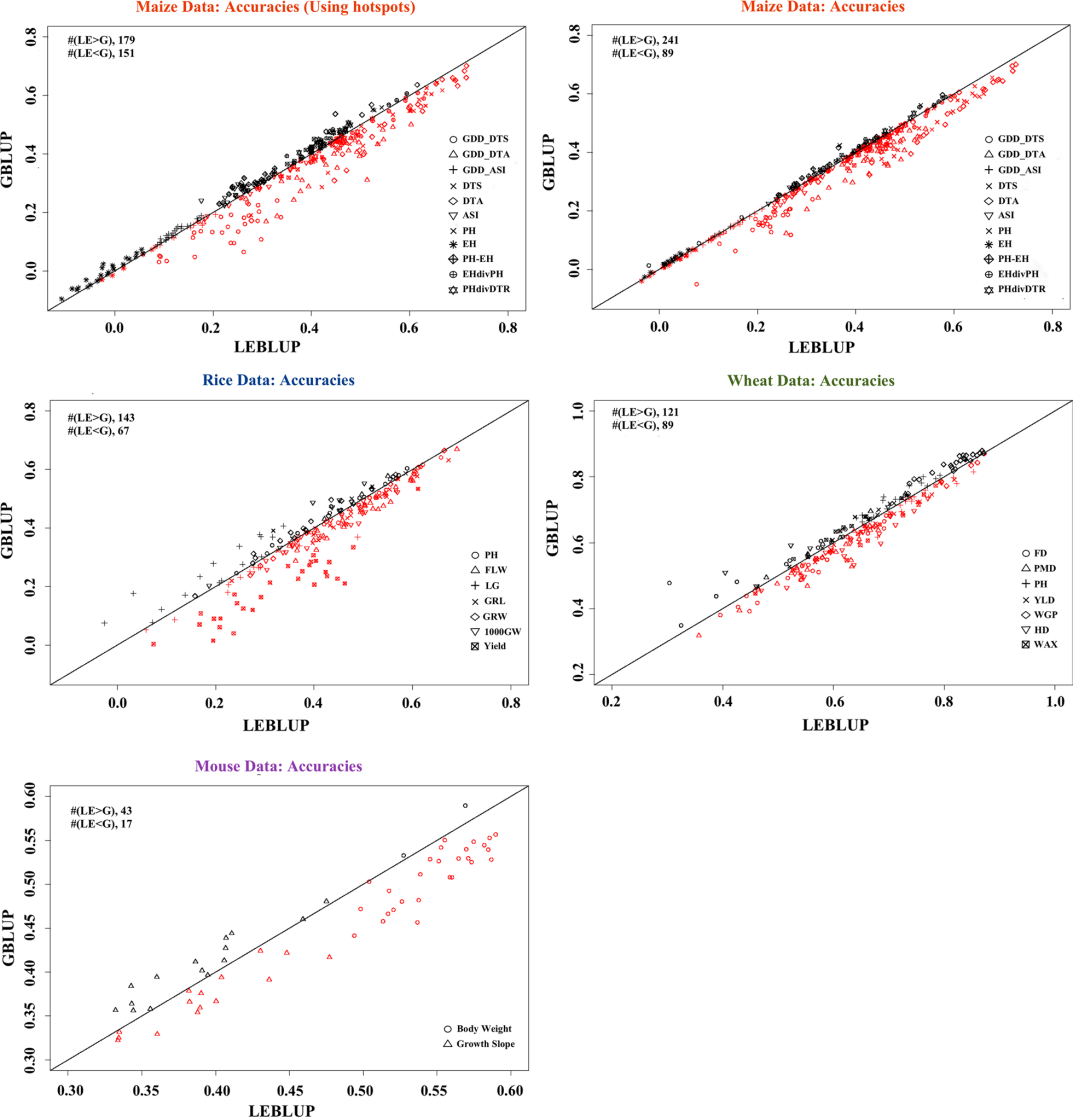
Accuracy obtained with the LER and GBLUP models (measured as the correlation between the estimated genetic values and the response variable) for each of the 30 replicates for each trait in all datasets. The red colored data points below the *y* = *x* show the instances where the LER models performed better than the GBLUP models. Black colored data points show the instances where the GBLUP models performed better than the LER models. The number of times that each model performed better than the other is shown on the top left side for each dataset. *GDD_DTA*: growing degree days to silk, *GDD_DTA* growing degree days to anthesis, *GDD_ASI* growing degree days to anthesis-silking interval, *DTS* days to silking, *DTA* days to anthesis, *ASI* anthesis silking interval days, *PH* plant height, *EH* ear height, *PH-EH* PH minus EH, *EHdivPH* EH divided by PH, *PHdivDTR* PH divided by days to anthesis *FLW* flag leaf width, *LG* lodging, *GRL* grain length, *GRW* grain weight, *1000GW* thousand grain weight, *YLD* yield, *FD* flowering day, *PMD* physiological maturity day, *WGP* whole grain protein, *HD* heading date Julian, *WAX* waxiness

**Fig. 4:**
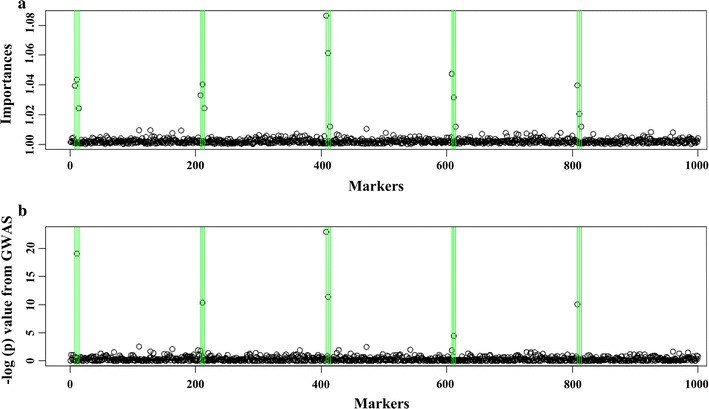
Importance scores from the LER model using the trait values and the genotypes generated as described in Table 3 based on a standard additive GWAS mixed model. The green lines highlight the SNPs that were used to calculate the genetic values. The importance scores and the results from the standard GWAS were similar. More SNPs were identified correctly as important by the LER approach

**Fig. 5:**
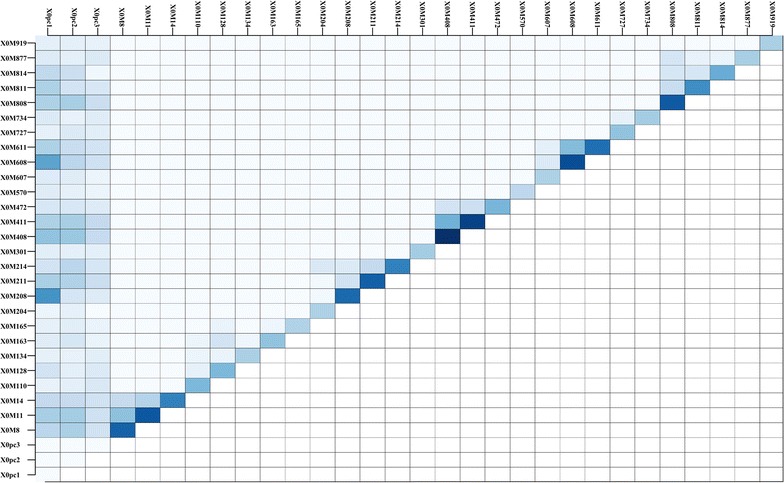
Additive and two-way interaction importance measures for the first three principal components and the 27 most important SNPs for one simulation as described in Table 3. The main effects of the SNPs are displayed on the diagonal and the off-diagonal shows two way interactions. The darker cells indicate more important SNPs, or interactions

 Figure 3 shows the accuracies obtained with the LER and GBLUP models for each of the 30 replicates for each trait in all datasets. The red colored data points show the cases for which the LER models performed better than the GBLUP models. The number of times that each model performed better than the other is shown on the top left side for each dataset. The performance for each trait can be evaluated also from the same figure using the legends. In general, the LER models performed better than GBLUP for all datasets and particularly well for traits with a complex genetic architecture, i.e., generated by a large number of genes with small effects, e.g. in the maize dataset, for growing degree days to anthesis (*GDD_DTA*) and to silking (*GDD_DTA*), anthesis-silking interval and plant height, yield components and in the mouse dataset for body weight. The results in Fig. 3 correspond to the hyper-parameter settings provided in Table 1. In Additional file 1: Figures S1 and S2, we provided the accuracies obtained for several other hyper-parameters settings. These results show that the hyper-parameter settings have a strong influence on the performance of LER models. Nevertheless, the gains in accuracies across the traits mentioned above are persistent for a wide range of hyper-parameter values. Figures 4 and 5 and Table 4 show the results from the simulation experiment that was described in the Methods section and in Table 4. In Fig. 4, we present one example of the association results for the ordinary GWAS approach using a mixed model and the importance scores obtained from the LER model. In this figure, the vertical blue lines show the simulated QTL. A comparison indicates that the LER model can identify QTL that are missed in the additive GWAS (Fig. 4). Figure 5 also displays one example of the importance and interaction statistics for the first three PC and 27 SNPs that are deemed important by the importance statistic. The main effects of the SNPs are shown on the diagonal and the off-diagonal shows two-way interactions. The darker the colors, the more important are the effects. According to Table 3, this figure shows that the LER model captured the simulated additive and interaction effects. For example, ×8, ×11 and ×14 SNPs have additive simulated effects (Table 3) and they do not interact. The second genetic effects 208, 211 and 214 interact with the background (PC1) but have additive effects. In both cases, the LER model captured the simulated effects. In addition, for 100 independent replications of the same simulation experiment, Table 3 provides the counts of the number of times each of the 15 SNPs that generate a genetic value appears in the top 20 SNPs selected by LER versus by additive GWAS. The results also showed that LER is superior in identifying QTL, especially when interaction effects are involved (Additional file 2).

## Discussion

This paper is an extension of a previous paper [40], in which we had defined a RKHS approach to capture epistasis between closely-linked markers (local epistasis) and we weighted the local estimates using an elastic net algorithm. Here, we propose a different approach that uses ensemble rules with the aim of capturing more complex interactions between predefined subsets of SNPs. First, the procedure extracts the locally epistatic effects using an ISLE ensemble rule and, then they are included into a standard random regression BLUP approach. The procedure is applied to simulated data and to four different datasets with satisfactory results when compared with GWAS or GBLUP. The focus of this article is on building locally epistatic models using rule ensembles. However, LER model building is a general methodology that includes three stages:

1. Divide the genome into regions.
2. Extract locally epistatic effects: Use the training data to obtain a model to estimate the locally epistatic effects.
3. Process locally epistatic effects by combining them using an additive model.

At each of these model building stages, the researcher needs to make a number of decisions. For example, in all of our implementations of the LER models, we have used non-overlapping contiguous regions. Nevertheless, the regions used in locally epistatic models can be overlapping or hierarchical. If some SNPs are associated with each other in terms of linkage or function, as for example through a known biochemical pathway, it might be useful to combine them together. For instance, the whole genome can be divided physically into chromosomes, chromosome arms or linkage groups. Further divisions could be based on recombination hot-spots or just merely based on local proximity. We can also group SNPs based on their effects on intermediate traits such as lipids, metabolites, or gene expression. With the development of next-generation sequencing and genotyping approaches, large haplotype datasets are becoming available in many species. These haplotype frameworks provide substantial statistical power in association studies of common genetic variation across each region. The locally epistatic framework can be used to take advantage of annotations of the variants relative to the genes they are in or their predicted impact on protein function. It is possible to build LER models where each SNP defines a region by its neighborhood. This definition would give overlapping regions. We can supplement the rules with the SNP scores, and then the model fitting procedure will provide the appropriate coefficients for the rules and the linear terms. After extracting rules from a region, a variable selection procedure can be applied to pick the most relevant rules from that region. A regression of the response variable on the set of rules from a region using the elastic-net loss function allows us to control the number of rules selected as relevant for that region. In particular, the elastic-net algorithm uses a loss function that is a weighted version of lasso and ridge-regression penalties. If all the weight is put on the ridge-regression penalty, no selection will be applied to the input variables. On the other extreme, if all the weight is put on the lasso penalty this will give maximal sparsity. We have treated this parameter as a hyper-parameter. The remaining parameters of the elastic-net regression were selected using cross-validation. In some cases, a very large rule ensemble is required to obtain a competitive discrimination between signal and background and to obtain reliable importance statistics. When the number of rules extracted from the data is too large to handle then the relationship in Eq. (9) can be used to obtain the rule effects. If environmental covariates are observed along with the trait values then it is possible to include these variables with the SNPs in each region while extracting the rules. This will allow environmental main effects + gene-by-environment interaction terms to enter the model. Variables that measure background genetic variability related to the structure of the population can be incorporated into the model in the same way. In the examples below, we used the first three principal components of the marker matrix along with the marker matrix to account for the genome-wide structural effects and the gene interactions. As mentioned previously, the best settings for the model, as determined by the best generalization performance, can be estimated via cross-validation or other model selection criteria for each model fitting instance. These settings, in turn, might be indicative of the trait architecture. For example, increasing the “mean depth” parameter in the wheat dataset to allow higher order interactions deteriorated the model performance and this can be taken as an indication that for this dataset genetic effects are additive or interactions are of low order. Whereas, for the rice dataset, the best settings for the model have relatively high “mean depths”, possibly indicating that in addition to additive effects, there are high levels of gene-by-gene, and gene-by-background interactions in this dataset. We also presented accuracy results for some other settings of the hyper-parameters of the LER algorithm in Additional file 1. The results of the simulated association experiment show that the importance and the interaction scores can be used to identify interesting loci. The comparisons with the standard additive mixed model GWAS showed that the LER methodology was superior: it detected loci that were not detected by the mixed model and at the same time provided a measure of the interactions between different types of input variables. We were able to recover most gene-by-gene and gene-by-background interactions with the LER model. We also described how this methodology can be used to study other forms of interaction. Finally, we highlighted some other strengths that are specific to the LER models:

- The method can incorporate SNP annotations if they are used to partition predictors into “regions”.
- Importance scores for regions, SNPs, and rules are available as a model output.
- The need to impute the SNP data is reduced: the model is robust to missing observations in the dataset.
- Marker-by-marker interactions and even higher order interactions can be captured and interaction statistics are also available as a model output.
- The model can be used to capture gene-by-genetic background or gene-by-environment interactions.

## Conclusions

In this paper, we analyzed four real datasets over many traits, and also provided results from a simulation study. For most traits, accuracy gains using the LER model were consistent regardless of the hyper-parameter values, e.g. several traits for the rice dataset, body weight for the mouse dataset, and days to anthesis for the maize dataset. We hypothesize that the LER model outperforms the additive model when the trait architecture involves local epistasis and gene–background interactions. For instance, the rice dataset has more family structure than the wheat dataset and it is reasonable to expect more gene-by-genetic background interactions in the former case. This could explain the differences in accuracy results between the additive GBLUP model and the LER model. We describe a new strategy for incorporating genetic interactions into genomic prediction and association models. This strategy results in accurate models, sometimes doubling the accuracies that can be obtained by a standard additive model.

## Additional files

**Additional file 1. Table S1:** Hyper-parameter settings for the results presented in Figure S1. **Table S2**: Hyper-parameter settings for the results presented in Figure S2. **Figure S1**: Accuracies for the rice dataset. **Figure S2**: Accuracies for the wheat dataset

**Additional file 2.** R code for replicating the simulated experiment.
