# Supplementary material for: Locally epistatic models for genome-wide prediction and association by importance sampling
Source: Genet Sel Evol. 2017 Oct 17;49:74. doi: 10.1186/s12711-017-0348-8 (PMC5646165; doi:10.1186/s12711-017-0348-8)

Additional File 1

Deniz Akdemir

November 17, 2016

Table S1: Hyper-parameter settings for the results presented in Figure S1.

nsplit=5, meandepth=2	nsplit=10, meandepth=2
nsplit=20, meandepth=2	nsplit=5, meandepth=3
nsplit=5, meandepth=4	nsplit=10, meandepth=3

Table S2: Hyper-parameter settings for the results presented in Figure S2.

nsplit=3, meandepth=1	nsplit=5, meandepth=1
nsplit=5, meandepth=2	nsplit=2, meandepth=1
nsplit=2, meandepth=2	nsplit=2, meandepth=3

Figure S1: The accuracies (measured as the correlation between the estimated genetic values and the response variable) for GBLUP and the LER model compared for the rice dataset for a few different hyper-parameter settings. The red points below the $y = x$ line are the cases where the LER model was more accurate than GBLUP.

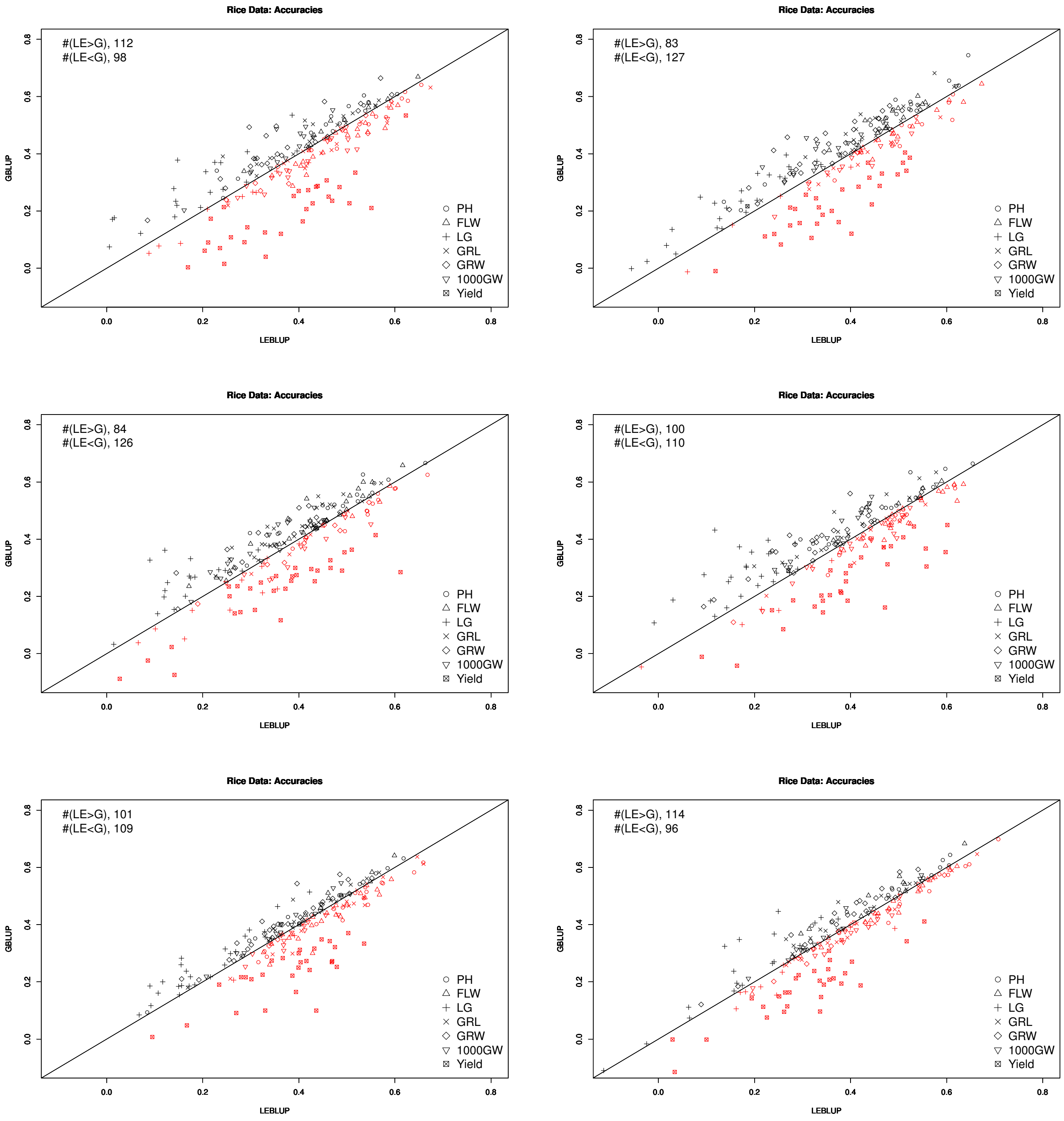

Figure S2: The accuracies (measured as the correlation between the estimated genetic values and the response variable) for GBLUP and the LER models compared for the wheat dataset for a few different hyper-parameter settings. The red points below the $y = x$ line are the cases where the LER model was more accurate than GBLUP.

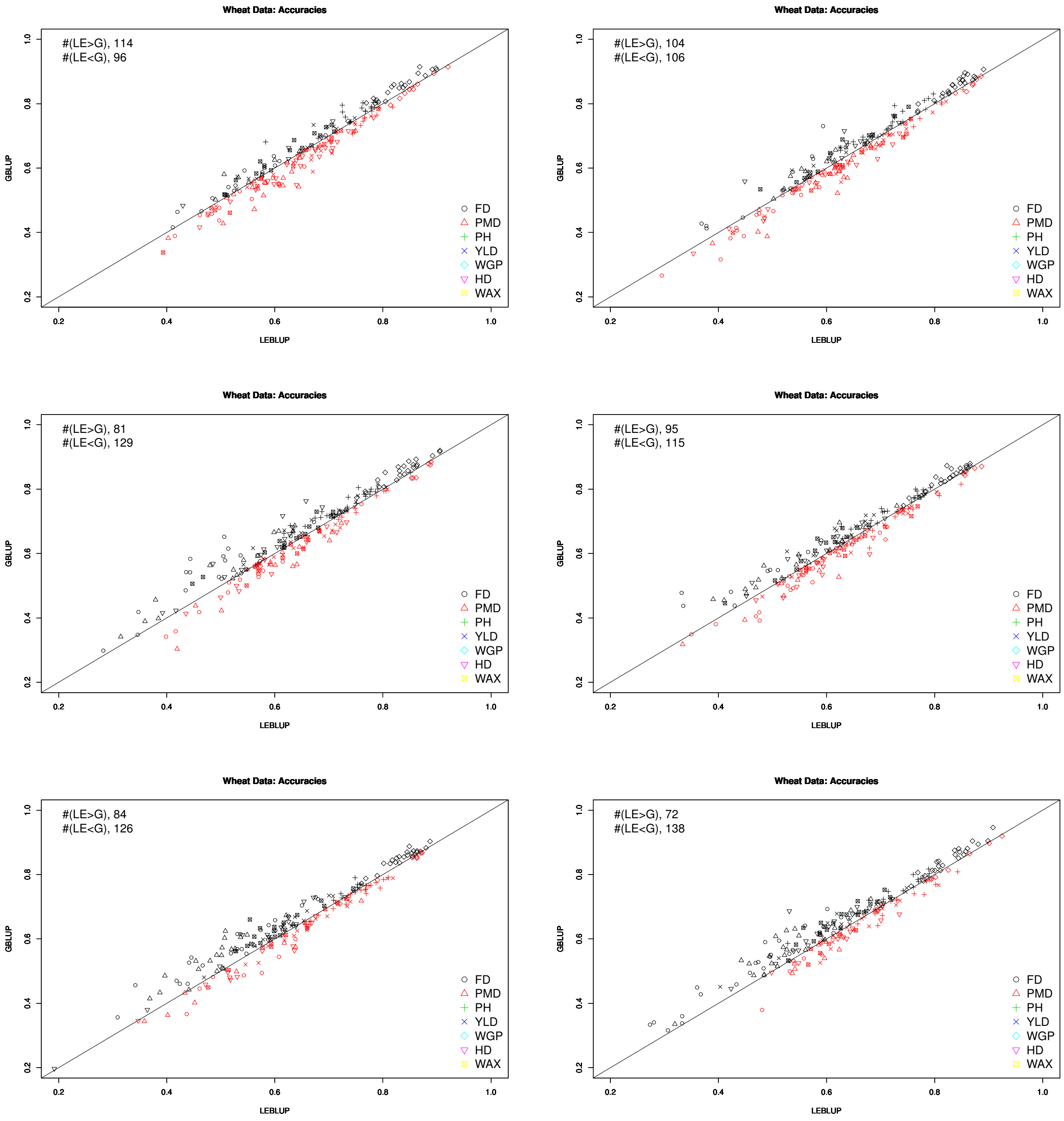

Supplement: Supplementary file 1 — Additional file 1. Table S1: Hyper-parameter settings for the results presented in Figure S1. Table S2: Hyper-parameter settings for the results presented in Figure S2. Figure S1: Accuracies for the rice dataset. Figure S2: Accuracies for the wheat dataset [file 12711_2017_348_MOESM1_ESM.pdf]
